# Sprint interval training decreases left-ventricular glucose uptake compared to moderate-intensity continuous training in subjects with type 2 diabetes or prediabetes

**DOI:** 10.1038/s41598-017-10931-9

**Published:** 2017-09-05

**Authors:** Marja A. Heiskanen, Tanja J. Sjöros, Ilkka H. A. Heinonen, Eliisa Löyttyniemi, Mikko Koivumäki, Kumail K. Motiani, Jari-Joonas Eskelinen, Kirsi A. Virtanen, Juhani Knuuti, Jarna C. Hannukainen, Kari K. Kalliokoski

**Affiliations:** 1 Turku PET Centre, University of Turku, Turku, Finland; 2 Department of Clinical Physiology and Nuclear Medicine, University of Turku and Turku University Hospital, Turku, Finland; 3 Department of Biostatistics, University of Turku, Turku, Finland

## Abstract

Type 2 diabetes mellitus (T2DM) is associated with reduced myocardial glucose uptake (GU) and increased free fatty acid uptake (FFAU). Sprint interval training (SIT) improves physical exercise capacity and metabolic biomarkers, but effects of SIT on cardiac function and energy substrate metabolism in diabetic subjects are unknown. We tested the hypothesis that SIT is more effective than moderate-intensity continuous training (MICT) on adaptations in left and right ventricle (LV and RV) glucose and fatty acid metabolism in diabetic subjects. Twenty-six untrained men and women with T2DM or prediabetes were randomized into two-week-long SIT (n = 13) and MICT (n = 13) interventions. Insulin-stimulated myocardial GU and fasted state FFAU were measured by positron emission tomography and changes in LV and RV structure and function by cardiac magnetic resonance. In contrast to our hypothesis, SIT significantly decreased GU compared to MICT in LV. FFAU of both ventricles remained unchanged by training. RV end-diastolic volume (EDV) and RV mass increased only after MICT, whereas LV EDV, LV mass, and RV and LV end-systolic volumes increased similarly after both training modes. As SIT decreases myocardial insulin-stimulated GU compared to MICT which may already be reduced in T2DM, SIT may be metabolically less beneficial than MICT for a diabetic heart.

## Introduction

Type 2 diabetes mellitus (T2DM) is a strong risk factor of cardiovascular morbidity and mortality, and it increases the risk of developing heart failure even without co-existence of coronary artery disease or hypertension^1–4^. Diabetic cardiomyopathy is characterized in asymptomatic patients by left ventricular (LV) diastolic dysfunction^5^, followed by increased LV mass and wall thickness^6^. Eventually, also LV systolic dysfunction may occur, potentially leading to heart failure^7^. Corresponding functional impairments have been reported also regarding the right ventricle (RV)^8, 9^.

Alterations of myocardial metabolism play a major role in the development of diabetic cardiomyopathy^10, 11^. In a healthy heart, ~70% of ATP required in fasted state is produced by oxidation of fatty acids and ~30% by glucose and lactate, and the heart can rapidly switch its substrate utilization in response to changes in physiological or pathophysiological conditions. However, in a diabetic heart fatty acid oxidation is increased^12, 13^ while glucose utilization and flexibility in substrate use are often decreased^13–17^, although also normal myocardial glucose uptake values have been reported^18, 19^. Continuous high fatty acid uptake increases not only fatty acid intermediates within the heart muscle, but also oxygen consumption and generation of reactive oxygen species, which are believed to cause myocardial damage and contractile dysfunction^10, 12^.

Regular exercise training can modify the risk of T2DM^20^ and may also alleviate the metabolic abnormalities of a diabetic heart^21^. Current guidelines of the American Diabetes Association recommend at least 150 min of moderate intensity (40–60% of maximal oxygen uptake VO_2max_) or 75 min of vigorous intensity exercise (>60% of VO_2max_) per week for patients with T2DM^20^. However, many people fail to achieve this amount of exercise, commonly citing lack of time as a barrier^22^. Therefore, high-intensity interval training (HIIT), as well as “all-out” sprint interval training (SIT), have gained interest as more time-efficient methods of exercise compared to traditional moderate-intensity continuous training (MICT)^23^. HIIT effectively improves cardiorespiratory fitness and glucose control in subjects with T2DM or metabolic syndrome^24, 25^. However, less is known about the effects of HIIT or SIT on cardiac function and metabolism of a diabetic heart, although previous studies in subjects with T2DM have suggested that HIIT can improve LV function compared to non-exercising controls^26^ or MICT^27^.

In the present randomized controlled trial, we compared the effects of two weeks of SIT and MICT on both the LV and RV glucose uptake (GU) and free fatty acid uptake (FFAU) in subjects with T2DM or prediabetes using positron emission tomography (PET). As secondary outcomes, myocardial perfusion in LV was measured by PET, and changes in LV and RV structure and function were studied by cardiac magnetic resonance (CMR). We hypothesized that exercise training would increase insulin-stimulated GU and decrease fasting FFAU in subjects with T2DM or prediabetes, and that SIT would be more effective to induce these adaptations compared to MICT. Finally, we expected that SIT would improve LV and RV function more than MICT.

## Methods

### Study design

**Figure 1:**
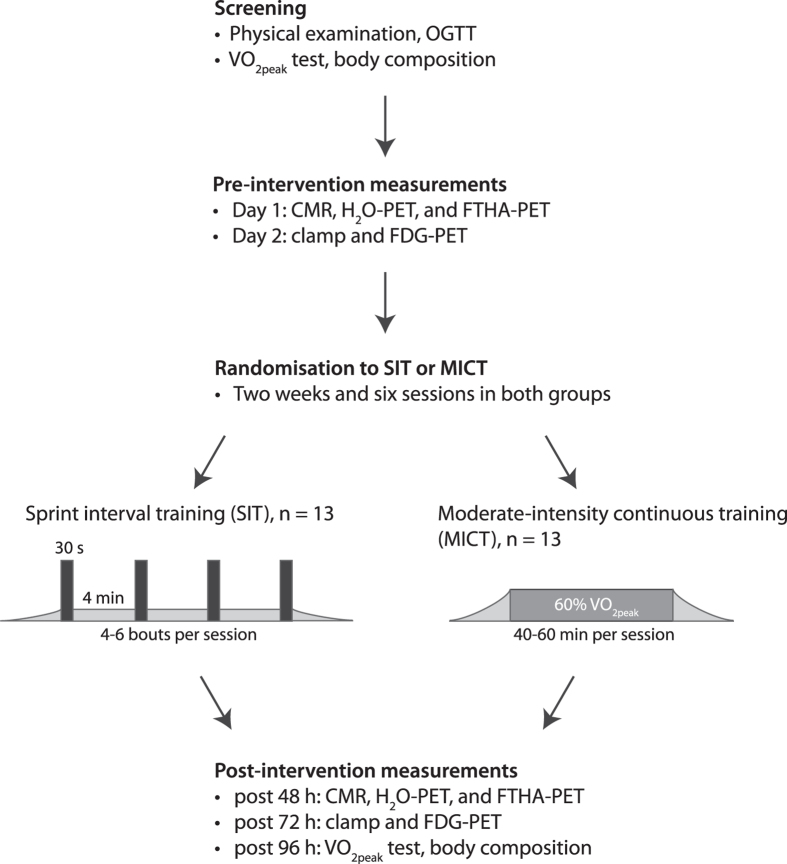
Study design. OGTT, oral glucose tolerance test; VO_2peak_, peak oxygen uptake; CMR, cardiac magnetic resonance; FTHA-PET, positron emission tomography study for myocardial fat metabolism; FDG-PET, positron emission tomography study for myocardial glucose metabolism.

This study was a parallel-group randomized controlled trial with 1:1 allocation ratio conducted at Turku PET Centre (Turku, Finland) between February 2013 and October 2015. The study flow is illustrated in Fig. 1. The present study is a part of a larger study titled “*The Effects of Short-Term High-Intensity Interval Training on Tissue Glucose and Fat Metabolism in Healthy Subjects and in Patients with Type 2 Diabetes*” (NCT01344928, date: April 28, 2011). The study was conducted according to Declaration of Helsinki, and the study protocol was approved by the ethical committee of the Hospital District of Southwest Finland, Turku (decision 95/180/2010 §228). Written informed consent was obtained from all subjects before the beginning of the study.

### Subjects

The study included subjects with relatively newly diagnosed T2DM or prediabetes that could benefit from an exercise training intervention. The participants were recruited with advertisements in local newspapers, through personal contacts, and using electronic and traditional bulletin boards. Before the study, subjects were interviewed and thoroughly examined by a medical doctor, including ECG and oral glucose tolerance test (OGTT). A candidate was excluded if they had a condition which could potentially endanger subject’s health during the study or interfere with the interpretation of the results^28, 29^. A candidate was accepted to the study if following criteria were fulfilled: age 40–55 years (corresponding to age range when T2DM is often diagnosed), body mass index 18.5–35 kg∙m^−2^, blood pressure ≤ 160/100 mmHg, no exercise on regular basis (peak oxygen uptake VO_2peak_ ≤ 40 ml∙kg^−1^∙min^−1^), and defective glucose tolerance according to the criteria of the American Diabetes Association^30^ and HbA_1c_ less than 7.5 mmol/l. Of 57 screened subjects, 26 participants fulfilled the inclusion criteria and were admitted into the study. Criteria of T2DM^30^ were met in 17 subjects and 13 of them were treated with at least one type of oral hypoglycaemic agent (median T2DM duration 4 years), whereas 4 of them had no previous medication for T2DM. The remaining 9 subjects met the criteria of prediabetes, having impaired fasting glucose and/or impaired glucose tolerance^30^.

Randomization for SIT and MICT was performed with random permuted blocks of four subjects with 1:1 allocation ratio, resulting in n = 13 in SIT and n = 13 in MICT group. Subjects were informed about the groups they belonged to after the screening. Two subjects from the SIT group dropped out during the trial, one because of claustrophobic feelings in CMR during pre-intervention scan and one due to migraine during the first SIT session. Three subjects from the MICT group discontinued the trial due to personal reasons. Hence, 11 subjects in SIT and 10 subjects in MICT group finalized all their assigned training sessions and underwent follow-up measurements. The observer analyzing the PET and CMR images was blinded for the group allocation.

### Training interventions

The training interventions consisted of six exercise sessions within two weeks^28, 31–34^. Each session was performed in supervised laboratory conditions. The SIT training consisted of 4–6 × 30 s of all-out cycling efforts with 4 min of recovery (Monark Ergomedic 894E, Monark, Vansbro, Sweden). The number of bouts was increased from 4 to 5, and further to 6 after every other session. The participants were familiarized with SIT during the screening phase (2 × 30 s bouts). Each bout started with 5-second acceleration to maximal cadence without any resistance, followed by a sudden increase of the load (10% of fat free mass in kg) and maximal cycling for 30 s. The MICT group cycled (Tunturi E85, Tunturi Fitness, Almere, The Netherlands) for 40–60 min at the intensity of 60% of peak workload. The duration of cycling was increased from 40 min to 50 min, and further to 60 min after every other session.

### Outcome measures

Full details of the protocols used to determine the outcome measures of this study have been described elsewhere^29, 31, 32^ and are briefly summarized here. The number of completed and uncompleted experiments for the outcome measures are detailed in Table 1.

**Table 1:** Numbers of completed and uncompleted experiments in the study.

	SIT		MICT	
	Pre	Post	Pre	Post
Number of subjects who participated at least in one measurement	13	11	13	10
Myocardial GU, completed	11*	9	13	10
Missing because				
- discontinued intervention	1^†^	2	—	3
- technical problem in clamp	1	—	—	—
- physiological problem	—	1^‡^	—	—
- PRE-measurement was not done	—	1	—	—
Myocardial FFAU, completed	12	8	13	9
Missing because				
- discontinued intervention	—	2	—	3
- technical problem in tracer production	1	2	—	1
- PRE-measurement was not done	—	1	—	—
Basal perfusion, completed	13	10	13	10
Missing because				
- discontinued intervention	—	2	—	3
- technical problem in tracer production or PET scanning	—	1	—	—
Adenosine-stimulated perfusion, completed	11	9	13	10
Missing because				
- discontinued intervention	—	2	—	3
- physiological problem	2^§^	2^§^	—	—
CMR, completed	12	11	13	10
Missing because				
- discontinued intervention	—	2	—	3
- physiological problem	1^†^	—	—	—

SIT, sprint interval training; MICT, moderate-intensity continuous training; GU, glucose uptake; FFAU, free fatty acid uptake; CMR, cardiac magnetic resonance imaging.

*For RV GU, number of analysed subjects was 10 due to poor visibility of the RV free wall in one subject.

^†^Subject experienced claustrophobic feelings during CMR scan and dropped out from the trial after the first measurement day.

^‡^Problem in the cannulation.

^§^Two subjects had a history of asthma.

The primary outcome of the study was to determine the effects of SIT and MICT on LV and RV metabolism (GU and FFAU) using PET. The PET imaging was performed with GE Advance PET/CT scanner (General Electric Medical System, Milwaukee, WI, USA). FFAU was studied at fasted state using 14(*R,S*)-[^18^F]fluoro-6-thia-heptadecanoic acid ([^18^F]FTHA; 155 (SD 9) MBq) as a tracer. On a separate day, GU was measured using 2-deoxy-2-[^18^F]fluoro-D-glucose ([^18^F]FDG; 157 (SD 10) MBq) during euglycemic hyperinsulinemic clamp. PET image raw files were corrected for attenuation, dead time, and decay. Images were reconstructed using 3D-OSEM procedure and analyzed using Carimas software (version 2.9, www.turkupetcentre.fi/carimas). Regions of interest (ROIs) were defined semi-automatically for LV, including the septum^32^. For RV, ROIs were drawn manually over the entire RV free wall^31^. Fractional tracer uptake rate was calculated from tissue and plasma time activity curves by graphical analysis. GU and FFAU were obtained by multiplying the fractional tracer uptake rate with the plasma glucose or free fatty acid (FFA) concentration during the scanning, respectively.

As secondary outcome, myocardial perfusion in LV was studied by PET using [O^15^]H_2_O as the tracer as described in detail previously^32^. The measurements were performed in the basal state as well as during intravenous adenosine infusion^35, 36^, and perfusion reserve was calculated as perfusion during adenosine infusion divided by basal perfusion. Basal and adenosine-stimulated coronary resistances were calculated by dividing mean arterial pressure measured during the scans with basal and adenosine-stimulated perfusion, respectively. LV blood flow was calculated with the single-compartment model using Carimas software^32^. As there is currently no validated model for the calculation of RV perfusion, we do not report RV perfusion in this study.

Also as secondary outcomes, exercise-induced adaptations of LV and RV structure and function was assessed by CMR using a Philips 1.5T Gyroscan Intera CV Nova Dual MR scanner (Philips Medical Systems, Best, The Netherlands) using previously described imaging parameters^29^. Image analysis was performed with Philips Extended MR WorkPlace version 2.6.3.5 (Philips Medical Systems, Best, The Netherlands) following the established guidelines^37^. Briefly, the endo- and epicardial contours were traced so that the papillary muscles and trabeculations were included in the LV and RV blood volumes, respectively. The interventricular septum was included in the LV mass and excluded from the RV analysis. Parameters derived from CMR included ejection fraction (EF), end-diastolic volume (EDV), end-systolic volume (ESV), stroke volume (SV), and mass for both ventricles. Cardiac output (CO) was calculated as the product of SV and resting heart rate, LV work as the product of LVCO and mean arterial pressure, and LV work index by dividing LV work by LV mass. Values normalized for body surface area (BSA) were calculated using the Dubois and Dubois formula^38^.

Body composition was measured by bioimpedance monitor (InBody 720, Mega Electronics Ltd., Kuopio, Finland). Whole-body insulin stimulated glucose uptake rate (M-value) was measured during euglycemic hyperinsulinemic clamp as described in detail previously^29, 33^. VO_2peak_ was determined by a maximal exercise test on a cycle ergometer (Ergoline 800 s; VIASYS Healthcare, Germany). As previously described^28, 29, 33, 34^, the test started at 50 W and the load was increased by 30 W at every 2 min until exhaustion. Ventilation and gas exchange was measured (Jaeger Oxycon Pro; VIASYS Healthcare) and reported as the mean value per minute. The peak respiratory exchange ratio was ≥ 1.17 and peak blood lactate concentration immediately and after 1 min was ≥ 7.4 mmol·l^−1^ for all the tests. The highest 1-min mean value of oxygen consumption was defined as VO_2peak_.

### Statistical analysis

Sample size was calculated for the whole study (NCT01344928) based on its primary outcome, skeletal muscle glucose uptake, as explained in detail by Sjöros *et al*.^34^. No sample size calculation was performed specifically on the outcome measures of the present study.

Normal distribution of the variables was tested using Shapiro-Wilk test and evaluated visually, and logarithmic transformations were performed when necessary. The difference between groups was tested by t-test for age and height and by Fisher’s exact test for the diagnostic group (T2DM/prediabetes), gender, and medications. The analyses for primary and secondary outcomes were carried out using intention-to-treat approach and hence, included all the randomized subjects. Statistical analyses were performed using hierarchical mixed linear model with compound symmetry covariance structure, including one within-factor (time; indicating overall mean change between baseline and measurement after intervention), one between-factor (group; SIT and MICT), and interaction term (group*time; indicating whether mean change during the study was different between the groups). Gender and diagnostic group (T2DM/prediabetes) were included as factors in all analyses. In addition, as plasma free fatty acid (FFA) concentration influences myocardial GU^39^, it was used as a covariate when analyzing LV GU and RV GU. Missing data points were accounted for by restricted maximum likelihood estimation within the linear mixed models. All statistical tests were performed as two-sided with statistical significance level set at 0.05. The analyses were performed using SAS System, version 9.3 for Windows (SAS Institute Inc., Cary, NC, US).

## Results

### Subject characteristics, glycemic control, and training efficacy

Based on the whole-body parameters (Table 2), the training groups were well matched at the baseline as described previously^34^. Body mass, BMI, and fat free mass remained unchanged after two weeks of training whereas fat percent mildly reduced (p = 0.018, time; Table 2). Fasting glucose, insulin, and 2 h glucose remained unaltered, but HbA_1c_ decreased after training (p = 0.002; Table 2). Whole-body insulin-stimulated glucose uptake (M-value) improved by both training modes (p = 0.001; Table 2). The response of VO_2peak_ was different between SIT and MICT (p = 0.0495, group*time; Table 2), and only SIT improved VO_2peak_ (p = 0.013 for time effect in SIT).

**Table 2:** Subject characteristics and whole-body responses to SIT and MICT.

	SIT		MICT		*P*		
	Pre	Post	Pre	Post	Group	Time	Group x Time
*n*	13	11	13	10			
men/women, *n*	9/4	7/4	7/6	6/4	0.688*		
T2DM/prediabetes, *n*	11/2	10/1	6/7	4/6	0.097*		
At least one glucose lowering medication, *n*	9		4		0.115*		
Metformin	7		4		0.428*		
DPP-4 inhibitors (sitagliptin)	4		1		0.322*		
Sulfonylurea (glimepiride)	1				0.999*		
Other medication, *n*							
Antihypertensives	5		6		0.999*		
Statins	4		3		0.999*		
Affective medication			3		0.220*		
Menopausal hormone therapy	1		2		0.999*		
Age, yr	49 (47, 51)		49 (46, 51)		0.849^†^		
Height, cm	173 (168, 179)		172 (167, 176)		0.614^†^		
Weight, kg	88.9 (80.6, 97.2)	88.4 (80.1, 96.7)	91.5 (84.5, 98.6)	91.1 (84.0, 98.1)	0.620	0.083	0.952
BSA, m^2^	2.0 (1.9, 2.1)	2.0 (1.9, 2.1)	2.0 (2.0, 2.1)	2.0 (1.9, 2.1)	0.543	0.131	0.877
BMI	30.5 (28.5, 32.5)	30.3 (28.4, 32.3)	31.0 (29.4, 32.7)	30.8 (29.2, 32.5)	0.688	0.070	0.833
Fat, %	34.8 (31.4, 38.5)	33.8 (30.5, 37.5)	33.8 (30.8, 36.9)	32.9 (30.0, 36.0)	0.666	**0.018**	0.872
FFM, kg	57.0 (51.8, 62.2)	57.6 (52.4, 62.8)	59.6 (55.0, 64.2)	59.8 (55.2, 64.5)	0.492	0.109	0.541
VO_2peak_, ml·kg^−1^·min^−1^	25.7 (23.2, 28.2)	27.0 (24.6, 29.5)^‡^	27.0 (24.9, 29.2)	26.9 (24.6, 29.1)^§^	0.716	0.123	**0.050**
M-value, µmol·kg^−1^·min^−1^	20.6 (13.4, 27.7)	25.7 (18.4, 33.0)	15.7 (9.7, 21.6)	19.7 (13.6, 25.8)	0.237	**0.001**	0.657
HbA_1c_, %	5.7 (5.4, 6.0)	5.5 (5.2, 5.8)	5.8 (5.5, 6.0)	5.6 (5.3, 5.9)	0.704	**0.002**	0.844
HbA_1c_, mmol/mol	38.7 (35.5, 42.0)	36.9 (33.6, 40.2)	39.6 (36.9, 42.4)	37.6 (34.7, 40.5)	0.701	**0.001**	0.816
Fasting glucose, mmol/l	7.1 (6.5, 7.7)	7.0 (6.4, 7.6)	6.8 (6.3, 7.3)	7.0 (6.4, 7.5)	0.715	0.953	0.395
OGTT 2 h glucose, mmol/l	10.4 (8.6, 12.3)	9.1 (7.2, 11.1)	10.5 (8.9, 12.1)	10.3 (8.6, 12.0)	0.604	0.089	0.207
Fasting insulin, pmol/l	11.5 (7.8, 17.0)	11.6 (7.8, 17.2)	13.2 (9.5, 18.5)	13.5 (9.5, 19.0)	0.565	0.881	0.933
OGTT 2 h insulin, pmol/l	75.0 (50.3, 111.7)	71.1 (46.6, 108.4)	68.1 (48.1, 96.3)	62.9 (42.7, 92.5)	0.654	0.615	0.921
Glucose AUC in OGTT	1274 (1142, 1407)	1242 (1104, 1379)	1298 (1184, 1412)	1323 (1200, 1446)	0.527	0.905	0.413
Insulin AUC in OGTT	7162 (5264, 9746)	7109 (5181, 9756)	6871 (5273, 8954)	6748 (5106, 8920)	0.810	0.849	0.937

The results are presented as means (95% CI) for age and height. For all other parameters the results are presented as model-based means (95% CI). Results have been reported previously by Sjöros *et al*.^34^. Group *p*-value indicates whether there is a level difference between the groups, time *p*-value displays the mean change between pre- and post-measurements and group x time *p*-value indicates whether the mean changes are different between the groups. SIT, sprint interval training; MICT, moderate-intensity continuous training; *n*, number of subjects; T2DM, type 2 diabetes mellitus; BSA, body surface area; FFM, fat free mass; AUC, area under curve; *Fisher’s exact test at baseline; ^†^T-test; ^‡^SIT time effect, p = 0.013; ^§^MICT time effect, p = 0.75. Significant differences are printed in boldface.

### Myocardial glucose and free fatty acid uptake and perfusion

In contrast to our hypothesis, SIT lowered insulin-stimulated LV GU by −6.0 μmol∙100 g^−1^∙min^−1^ (95% CI -12.0 to 0.1 μmol∙100 g^−1^∙min^−1^) whereas MICT increased it by 3.4 μmol∙100 g^−1^∙min^−1^ (95% CI -2.5 to 9.3 μmol∙100 g^−1^∙min^−1^). The difference between SIT and MICT was statistically significant (p = 0.030, group*time; Table 3), and the effect of SIT on LV GU tended to be statistically significant (p = 0.054; Table 3). While a similar pattern was observed for RV GU, the difference between the training protocols was not significant (p = 0.125; Table 3). Changes in LV GU did not correlate with the changes in FFAU, whole-body variables, or cardiac parameters in either of the groups.

**Table 3:** Myocardial metabolism and plasma concentrations during the PET studies and myocardial blood flow before and after SIT and MICT.

	**SIT**		**MICT**		***p***		
	**Pre**	**Post**	**Pre**	**Post**	**Group**	**Time**	**Group x Time**
**[** ^**18**^ **F]FDG-PET study**							
*Glucose metabolism*							
LV GU*, µmol·100 g^−1^·min^−1^	42 (35, 48)	36 (29, 43)	37 (32, 43)	41 (35, 47)	0.956	0.541	**0.030**
RV GU*, µmol·100 g^−1^·min^−1^	10.5 (7.6, 13.4)	9.4 (6.4, 12.4)	8.0 (5.7, 10.2)	8.7 (6.4, 11.0)	0.362	0.792	0.125
*Plasma concentrations*							
Glucose, mmol/l	4.7 (4.5, 4.9)	4.9 (4.7, 5.2)	5.0 (4.8, 5.2)	5.0 (4.8, 5.2)	0.117	0.122	0.241
FFA, mmol/l	0.09 (0.06, 0.11)	0.07 (0.05, 0.10)	0.09 (0.06, 0.11)	0.08 (0.05, 0.10)	0.931	0.126	0.902
Insulin, pmol/l	88.9 (80.3, 97.4)	90.4 (80.9, 99.8)	86.0 (78.8, 93.2)	85.8 (77.7, 94.0)	0.463	0.837	0.798
**[** ^**18**^ **F]FTHA-PET study**							
*Free fatty acid metabolism*							
LV FFAU, µmol·100 g^−1^·min^−1^	5.4 (4.4, 6.4)	5.7 (4.5, 6.8)	5.4 (4.6, 6.3)	5.2 (4.2, 6.2)	0.756	0.952	0.510
RV FFAU, µmol·100 g^−1^·min^−1^	2.0 (1.6, 2.4)	2.2 (1.8, 2.6)	2.2 (1.9, 2.5)	2.1 (1.7, 2.4)	0.935	0.750	0.248
*Plasma concentrations*							
Glucose, mmol/l	6.1 (5.6, 6.7)	5.9 (5.3, 6.5)	6.0 (5.5, 6.4)	5.9 (5.4, 6.4)	0.796	0.095	0.474
FFA, mmol/l	0.89 (0.77, 1.01)	0.87 (0.74, 1.01)	0.89 (0.79, 0.99)	0.86 (0.75, 0.97)	0.927	0.499	0.828
Insulin, pmol/l	10.8 (6.9, 14.6)	8.3 (4.3, 12.2)	10.4 (7.2, 13.5)	8.6 (5.3, 11.9)	0.993	**0.002**	0.539
**[O** ^**15**^ **]H**_**2**_ **O-PET study**							
Basal LV perfusion, ml·g^−1^·min^−1^	1.1 (1.0, 1.3)	1.2 (1.0, 1.4)	1.1 (1.0, 1.3)	1.1 (0.9, 1.2)	0.544	0.765	0.173
Adenosine-stimulated LV perfusion, ml·g^−1^·min^−1^	3.7 (3.0, 4.4)	3.9 (3.1, 4.7)	4.4 (3.8, 5.0)	4.7 (4.0, 5.4)	0.075	0.355	0.884
LV perfusion reserve	3.3 (2.6, 4.1)	3.4 (2.6, 4.2)	3.8 (3.2, 4.4)	4.4 (3.7, 5.1)	0.092	0.201	0.276
Basal coronary resistance, mm Hg·ml·g^−1^·min^−1^	93 (78, 108)	83 (67, 98)	99 (86, 112)	99 (85, 112)	0.244	0.161	0.192
Adenosine-stimulated coronary resistance, mm Hg·ml·g^−1^·min^−1^	26 (21, 32)	23 (18, 28)	25 (21, 29)	20 (16, 25)	0.407	0.055	0.775

The results are presented as model-based means (95% CI). Group *p*-value indicates whether there is a level difference between the groups, time *p*-value displays the mean change between pre- and post-measurements and group x time *p*-value indicates whether the mean changes are different between groups. SIT, sprint interval training; MICT, moderate-intensity continuous training; FDG, 2-deoxy-2-(18 F)fluoro-D-glucose; FTHA, 14(R,S)-[18 F]fluoro-6-thia-heptadecanoic acid; LV, left ventricle; RV, right ventricle; GU, glucose uptake; FFA, plasma free fatty acid concentration; FFAU, free fatty acid uptake. *Adjusted for plasma FFA concentration during the FDG-PET study. The FTHA-PET study was conducted in the fasting state and the FDG-PET study under hyperinsulinemic euglycemic clamp. Significant differences are printed in boldface.

FFAU of neither of the ventricles changed statistically significantly by training (Table 3). However, in the SIT group, changes in LV FFAU correlated negatively with changes in LV and RV end-diastolic volumes (r ≤ −0.72, p ≤ 0.045, both) and changes in RV FFAU correlated negatively with changes in LV EDV (r = −0.81, p = 0.014) and LV mass (r = −0.74, p = 0.035). Such correlations were not found in the MICT group.

The glucose and plasma FFA concentrations during both PET measurements were similar between the groups and did not change after the intervention (Table 3). The average of insulin levels during the FFAU measurements (fasting condition) decreased after training (p = 0.002, time) without difference between the groups (p = 0.99, group*time).

Both basal and adenosine-stimulated myocardial perfusion in LV were similar between the groups and they, together with perfusion reserve, remained unchanged by the training (Table 3). Basal coronary resistance remained unchanged, whereas adenosine-stimulated coronary resistance tended to decrease after training (p = 0.055, time) without difference in training response between the groups (p = 0.775, group*time).

### Cardiac dimensions and function and hemodynamic parameters

Both training modes increased LV EDV, LV ESV, and RV ESV (p < 0.020 all; Table 4). However, RV EDV responded differently to SIT and MICT (p = 0.022, group*time; Table 4), increasing only in MICT by 15 ml (95% CI 4 ml to 25 ml, p = 0.001 for time effect in MICT). Similarly, only MICT increased RV mass by 1.8 g (95% CI 0.2 g to 3.4 g, p = 0.036 group*time, p = 0.005 for time effect in MICT). LV mass slightly increased but not differently between the groups (p = 0.043 time, p = 0.813 group*time). All of these changes persisted also after normalizing for the BSA, and BSA-normalized parameters further highlighted the fact that the hearts in MICT group were larger compared to SIT group (Table 4). RV EF mildly decreased (p = 0.046, time) and LV EF tended to decrease after the training (p = 0.068, time) without a difference between the groups. Stroke volume and cardiac output of the both ventricles remained unchanged. However, BSA-normalized LV SV changed differently between the groups (p = 0.041, group*time). Finally, both SIT and MICT decreased all the hemodynamic parameters, including resting heart rate, blood pressure, and LV work index (Table 4).

**Table 4:** Cardiac dimensions and function and hemodynamic parameters before and after SIT and MICT.

	SIT		MICT		*p*		
	Pre	Post	Pre	Post	Group	Time	Group x Time
*LV dimensions and function*							
LV mass, g	100 (86, 113)	101 (87, 115)	120 (109, 132)	122 (111, 134)	**0.025**	**0.043**	0.813
LV mass/BSA, g/m^2^	49 (44, 54)	50 (45, 55)	59 (55, 63)	60 (56, 64)	**0.006**	**0.021**	0.809
LV EDV, ml	135 (115, 155)	140 (120, 160)	156 (139, 173)	166 (149, 183)	0.076	**<0.001**	0.153
LV EDV/BSA, ml/m^2^	67 (60, 73)	69 (63, 76)	76 (71, 82)	81 (76, 87)	**0.018**	**<0.001**	0.168
LV ESV, ml	48 (36, 60)	53 (41, 65)	56 (46, 66)	62 (51, 72)	0.276	**0.015**	0.952
LV ESV/BSA, ml/m^2^	24 (19, 28)	26 (21, 31)	27 (23, 31)	30 (26, 34)	0.210	**0.014**	0.958
LV SV, ml	87 (75, 98)	86 (75, 98)	100 (90, 109)	104 (94, 114)	**0.042**	0.147	0.054
LV SV/BSA, ml/m^2^	43 (39, 47)	43 (39, 47)	49 (46, 52)	51 (48, 54)	**0.010**	0.095	**0.041**
LV CO, l·min^−1^	6.3 (5.6, 7.0)	6.0 (5.2, 6.7)	6.8 (6.2, 7.4)	6.6 (5.9, 7.2)	0.215	0.108	0.663
LV CO/BSA, l·min^−1^/m^2^	3.2 (2.9, 3.5)	3.0 (2.6, 3.3)	3.2 (2.9, 3.5)	3.2 (2.9, 3.5)	0.490	0.331	0.223
LV EF, %	65 (62, 69)	63 (59, 67)	64 (61, 68)	63 (60, 67)	0.925	0.068	0.466
*RV dimensions and function*							
RV mass, g	24.8 (21.8, 27.8)	24.8 (21.8, 27.8)	27.5 (25, 30)	29.3 (26.7, 31.8)	0.069	**0.033**	**0.036**
RV mass/BSA, g/m^2^	12.3 (11.4, 13.2)	12.3 (11.4, 13.3)	13.4 (12.7, 14.2)	14.3 (13.5, 15.1)	**0.011**	**0.022**	**0.042**
RV EDV, ml	145 (126, 165)	147 (128, 167)	167 (151, 184)	182 (165, 199)	**0.030**	**0.004**	**0.022**
RV EDV/BSA, ml/m^2^	72 (66, 78)	74 (67, 80)	82 (76, 87)	89 (83, 95)	**0.004**	**0.002**	**0.028**
RV ESV, ml	59 (46, 72)	61 (48, 74)	69 (58, 79)	79 (68, 90)	0.092	**0.020**	0.120
RV ESV/BSA, ml/m^2^	29 (24, 34)	31 (25, 36)	33 (29, 38)	39 (34, 43)	0.069	**0.014**	0.149
RV SV, ml	86 (76, 97)	86 (75, 97)	99 (90, 108)	103 (93, 112)	**0.042**	0.138	0.094
RV SV/BSA, ml/m^2^	43 (39, 47)	43 (39, 47)	48 (45, 51)	50 (47, 53)	**0.010**	0.078	0.080
RV CO, l·min^-−1^	6.2 (5.6, 6.9)	5.9 (5.3, 6.6)	6.7 (6.2, 7.3)	6.4 (5.8, 7.1)	0.250	0.077	0.965
RV CO/BSA, l·min^−1^/m^2^	3.1 (2.8, 3.4)	3.0 (2.7, 3.3)	3.2 (2.9, 3.4)	3.2 (2.9, 3.5)	0.500	0.321	0.249
RV EF, %	60 (56, 64)	58 (54, 63)	60 (56, 63)	57 (53, 61)	0.721	**0.046**	0.688
*Hemodynamic parameters*							
HR_rest_, beats/min	73 (68, 78)	70 (65, 75)	69 (64, 73)	64 (59, 68)	0.076	**0.005**	0.376
BP_syst_, mm Hg	135 (129, 142)	133 (126, 140)	146 (141, 152)	139 (133, 145)	**0.043**	**0.010**	0.192
BP_diast_, mm Hg	86 (81, 90)	82 (77, 87)	89 (85, 93)	82 (77, 86)	0.652	**0.003**	0.354
BP_map_, mm Hg	102 (97, 107)	99 (93, 104)	108 (104, 112)	101 (96, 105)	0.194	**0.002**	0.269
RPP, mm Hg·min^−1^	9855 (9073, 10637)	9334 (8509, 10158)	10008 (9328, 10687)	8854 (8079, 9630)	0.736	**0.003**	0.211
LV work, mm Hg ·l·min^−1^	643 (558, 729)	587 (499, 675)	733 (660, 806)	662 (580, 743)	0.129	**0.017**	0.760
LV work index, mm Hg ·l·min^−1^·g^−1^	6.6 (5.9, 7.4)	6.0 (5.2, 6.8)	6.2 (5.6, 6.9)	5.6 (4.9, 6.3)	0.342	**0.005**	0.941

The results are presented as model-based means (95% CI). Group *p*-value indicates whether there is a level difference between the groups, time *p*-value displays the mean change between pre- and post-measurements and group x time *p*-value indicates whether the mean changes are different between groups. SIT, sprint interval training; MICT, moderate-intensity continuous training; LV, left ventricle; RV, right ventricle; BSA, body surface area; EDV, end-diastolic volume; ESV, end-systolic volume; SV, stroke volume; CO, cardiac output; EF, ejection fraction; HR_rest_, heart rate at rest; BP_syst_, systolic blood pressure; BP_diast_, diastolic blood pressure; BP_map_, mean arterial pressure; RPP, rate pressure product (calculated as HR_rest_·BP_syst_). The hemodynamic parameters describe the mean values of the screening day (included only in the pre-intervention values) and the two PET study days before and after the intervention. Significant differences are printed in boldface.

## Discussion

We compared the effects of short-term SIT and MICT interventions on both LV and RV metabolism and function in subjects with T2DM or prediabetes. Our hypothesis was that SIT would be effective in increasing insulin-stimulated GU and decreasing fasting FFAU, and this was considered to be beneficial adaptation to exercise. In contrast to our hypothesis, SIT decreased LV GU compared to MICT whereas FFAU remained unchanged by both SIT and MICT. While both training modes increased LV EDV, LV mass, and ESV of both ventricles and improved several hemodynamic variables, only MICT increased RV EDV and RV mass. The results suggest that SIT may be less beneficial than MICT for cardiac metabolism and function in subjects with T2DM or prediabetes, even though only SIT improved VO_2peak_.

The difference between the training modes was statistically significant only in LV GU although the same trend was observed in RV GU. While previous studies on the effects of exercise on myocardial GU in T2DM humans are lacking, both HIIT and MICT increased the rate of myocardial glucose oxidation in diet-induced obese mice^40^. This discrepancy may be due to several reasons, including different measurement techniques, health status of the study subjects, and the difference between animal model and humans. The observed difference between SIT and MICT could be due to differentially altered proportions of glucose and FFAs utilized as energy substrates. In addition to FFAs and glucose, lactate is an important energy substrate of the heart, especially during intense exercise when blood lactate concentration is increased. It has been shown that blood lactate at rest, as well as during insulin clamp, is higher in T2DM patients compared to healthy subjects^41^. In an animal study, 7 weeks of endurance training increased the expression of mitochondrial MCT1 in the hearts of diabetic rats, which is responsible for increasing lactate uptake^42^. Therefore, it is possible that intense all-out SIT stimulated the lactate uptake more than MICT and contributed to decreased GU.

Another explanation for the decreased LV GU by SIT compared to MICT could be increased LV mass leading to reduction of myocardial workload per gram of tissue, which was previously suggested to explain reduced LV GU in endurance athletes compared to controls^43^. In the present study LV mass was slightly increased and LV work index was decreased, but not differently between the training groups. Hence, reduction of LV work index does not likely account for the different responses of LV GU to SIT and MICT observed in these subjects.

The divergent response in glucose uptake to SIT and MICT in subjects with T2DM or prediabetes differs from our previous result on healthy subjects where both training modes reduced LV GU and RV GU^31, 32^. Although the decrease was not statistically significantly different between SIT and MICT, the reduction of GU seemed to be larger after SIT (−23% in LV and −22% in RV) compared to MICT (−6% in LV and −12% in RV) also in the healthy subjects^31, 32^. While both insulin-stimulated LV GU and RV GU seemed to be lower in subjects with T2DM or prediabetes in the present study compared to the healthy subjects at the baseline, after the training the difference diminished due to reduced GU of the healthy subjects. Previous studies have reported decreased LV GU in T2DM compared to healthy controls^13, 14^, while decreased RV GU has previously been reported only in Zucker diabetic fatty rats^44^. On the other hand, decreased LV GU per muscle volume unit has also been reported in athletes compared to sedentary controls^45^. Thus, both T2DM and exercise training in healthy subjects seem to lead to reduced insulin-stimulated myocardial GU. While the training-induced decrease in GU may be due to physiological hypertrophy and the heart muscle becoming more economical, the decreased GU in T2DM could be attributed to decreased number of sarcolemmal glucose transporters (GLUT4 and GLUT1) in diabetic cardiac cells^46^.

Intuitively, the decreased GU after SIT compared to MICT in subjects with T2DM or prediabetes, whose GU may be already lower compared to healthy subjects at baseline^13–16^, appears to be maladaptive rather than a positive change. Yet, several whole-body and circulatory variables improved similarly after SIT and MICT, including whole-body glucose uptake, HbA_1c_, resting heart rate, and blood pressure. Further, the well-established indicator of cardiovascular fitness, VO_2peak_, improved only after SIT. Therefore, further studies are needed to clarify whether the decrease of GU after SIT, both in healthy and diabetic subjects, is beneficial or harmful for the heart.

Training had no statistically significant effect on FFAU in subjects with T2DM or prediabetes. The result is in line with our previous study in healthy men, in which FFAU remained also unchanged after corresponding training interventions^31, 32^. However, both LV FFAU and RV FFAU seemed to be higher in subjects with T2DM or prediabetes compared to the healthy subjects. Previously, both HIIT and MICT decreased myocardial triglyceride content in diet-induced obese mice^40^, but it was unaltered by exercise training in human T2DM subjects^47, 48^. We recently showed that SIT tended to reduce myocardial triglyceride content compared to MICT in healthy and T2DM or prediabetic men^49^. However, no correlation was found between changes in myocardial triglyceride content and changes in FFAU or GU in the T2DM and prediabetic subjects of the present study (data not shown). On the other hand, treatment of T2DM with pioglitazone also had no effect on myocardial FFAU, even though pioglitazone improved LV EDV and LV SV^50^. It is believed that excessive lipid accumulation in the diabetic heart leads to contractile dysfunction^10, 12^, but it appears that cardiac function can be improved, at least to some extent, without a decrease in FFAU. However, in the SIT group we found statistically significant negative correlations between the changes of FFAU and changes in end-diastolic volumes of both ventricles and LV mass, implying that increase in EDV and LV mass may be related to even small decrease in FFAU. On the other hand, in the MICT group, EDV, ESV, and mass of the both ventricles increased independently to changes in FFAU.

Both basal and adenosine-stimulated LV perfusion remained unchanged after SIT and MICT. This can be considered as a positive finding as in our previous study on healthy men, adenosine-stimulated perfusion decreased significantly after SIT compared to MICT^32^. In addition, adenosine-stimulated coronary resistance tended to decrease after SIT and MICT in the present study, suggesting that training may have positive effect on coronary function in subjects with T2DM or prediabetes. These findings warrant further studies investigating the possible effects of longer training interventions on myocardial perfusion as well as studies investigating the mechanisms explaining the different training responses in healthy subjects and subjects with T2DM or prediabetes.

Both training modes increased LV EDV, LV ESV, and RV ESV similarly as in our previous studies in healthy subjects^31, 32^. As discussed previously^31, 32^, increase in cardiac volumes as a response to two weeks of exercise training is most likely related to training-induced increased plasma volume. Interestingly, only MICT increased RV EDV whereas SIT did not have significant effect on it. RV EF decreased mildly but statistically significantly and LV EF also tended to decrease after training. Decreased EF, both in RV and LV, is one feature of the diabetic cardiomyopathy^51^. However, the baseline ejection fractions in the subjects with T2DM or prediabetes in this study were comparable to those of the healthy men, and LV EF and RV EF decreased after the both training modes also in the healthy subjects^31, 32^. Further, RV EF has been shown to be smaller in elite athletes compared to nonathletes^52^. Therefore, the small reduction of EF observed in the subjects with T2DM or prediabetes is more likely a physiological response to training rather than pathophysiological change.

In regards to cardiac hypertrophy, there was a small increase in LV mass after both training modes, whereas only MICT increased RV mass. It has previously been stated that more than three hours of leisure-time sports in a week is required to increase LV mass^53^. However, more recent studies have shown approximately 12% increases in LV and RV masses after eight weeks of rowing^54^ or 12 weeks of HIIT on cycle ergometer^26^. It may be that technical advancements and increased sensitivity of imaging methodologies, especially CMR, allows detecting even small changes in cardiac structure. However, we did not observe changes in ventricular masses after two weeks of training in healthy subjects^31, 32^. Pathological hypertrophy is related to T2DM, but it is accompanied with reduced EDV. In our study, EDV increased and therefore the minor increase in cardiac mass is most likely a sign of positive, physiological hypertrophy rather than pathophysiological phenomenon.

Both exercise modes decreased several hemodynamic variables, including resting heart rate, systolic and diastolic blood pressure, rate pressure product, and LV work index. Further, both SIT and MICT increased whole-body insulin sensitivity and decreased HbA_1c_. Previous studies have reported decreased HbA_1c_ only after HIIT^26, 27, 55^ or no response in HbA_1c_^56^. Interestingly, VO_2peak_ improved only after SIT but remained unchanged by MICT. This result differs from our previous findings in healthy men in which both training modes improved VO_2peak_. However, the result is in line with the previous studies on T2DM or obese subjects in which HIIT has induced greater improvement in VO_2peak_ compared to MICT^24, 27, 55^.

The major strength of the study is the use of sophisticated methodology of positron emission tomography to measure the primary outcomes of the study, GU and FFAU, simultaneously in both ventricles. PET enables the quantitative analysis of myocardial substrate uptake, and to the best of our knowledge, this is the first time when metabolism of the RV is studied in subjects with T2DM or prediabetes. The secondary outcomes, namely structural and functional parameters of the heart, were measured with CMR, which is regarded as the golden standard in determination of these parameters^57^. All of the exercise sessions were performed in the supervised laboratory conditions. Finally, as the healthy sedentary men performed previously similar protocol, the responses of the subjects with T2DM or prediabetes of this study are comparable to our previous study^31, 32^ in the healthy subjects.

Nevertheless, the study is not without limitations. The subjects were both men and women, but the number of each gender was too small to address possible differences between genders, which may affect heart’s metabolic and functional responses to diabetic therapies^58^ as well as responses of VO_2peak_^59^. In addition, the subjects included both T2DM subjects with median T2DM duration of four years, as well as prediabetic subjects. Although the T2DM subjects were relatively newly diagnosed, the state of their disease may differ from that of prediabetic subjects, and it may have affected also the training responses. Further, there were somewhat uneven proportions of T2DM and prediabetic subjects in the SIT and MICT groups, though the difference was not statistically significant. Gender and diagnostic group (T2DM/prediabetes) were included as factors in the statistical analyses to minimize the effects of these confounding factors. As there were more T2DM subjects and the CMR-derived cardiac dimensions were smaller in the SIT group than in the MICT group, it is possible that the SIT group may have been more affected by diabetic cardiomyopathy. Further, it is possible that different medications may have interfered with the effects of training, although the use of medications was fairly similarly distributed in both groups. Nevertheless, metformin has been shown to reduce metabolic rate of glucose uptake in T2DM patients^50^, and we cannot completely rule out the possibility that medications contribute to the observed difference in LV GU after SIT and MICT. However, the study subjects represent typical T2DM and prediabetic patients with different medications for T2DM as well as often co-existing hypertension and hyperlipidemia, and therefore the study population reflects a real-life situation. Oral hypoglycaemic medications were interrupted for two days before the pre- and post-measurement PET scans, but it was not ethical or even clinically possible to prohibit the use of the prescribed drugs during the entire trial. Finally, the present study was designed to investigate the early-phase responses to SIT and MICT. Further studies with longer duration exercise interventions are needed to clarify, for instance, whether the decrease of GU in a response to SIT is only acute response to demanding all-out exercise, or will it remain decreased also by prolonged period of SIT.

## Conclusion

This study suggests that SIT may be less beneficial for a diabetic heart than MICT when considering exercise for subjects with T2DM or prediabetes. While SIT improved the exercise capacity of T2DM and prediabetic subjects, it decreased the myocardial GU compared to MICT. As the myocardial GU may already be reduced in T2DM, it is unclear whether SIT is a good treatment strategy for a diabetic heart. Therefore, further studies with longer duration exercise interventions are needed to investigate the mechanisms behind the decreased GU after SIT to determine whether it is a physiological or pathophysiological phenomenon.
